# Cortical thickness in the intertrochanteric region may be relevant to hip fracture type

**DOI:** 10.1186/s12891-017-1669-z

**Published:** 2017-07-18

**Authors:** Huafeng Zhuang, Yizhong Li, Jinkuang Lin, Donglu Cai, Siqing Cai, Lisheng Yan, Xuedong Yao

**Affiliations:** 1 Department of Orthopedics, the Second Affiliated Hospital of Fujian Medical University, Quanzhou, Fujian 362000 China; 2 Department of Radiology, the Second Affiliated Hospital of Fujian Medical University, Quanzhou, Fujian 362000 China

**Keywords:** Bone mineral density, Geometry, Hip fracture, Osteoporosis, DXA

## Abstract

**Background:**

This study assessed the differences in femoral geometry and bone mineral density between femoral neck fragility fractures and trochanteric fractures.

**Methods:**

One hundred and seventeen patients were divided into femoral neck and trochanteric fracture groups. There were 69 patients with femoral neck fractures, 20 men and 49 women with an average age of 75.1 ± 9.6 years and an average body mass index (BMI) value of 21.6 ± 4.1 kg/m^2^. The trochanteric group consisted of 48 patients, 16 men and 32 women with an average age of 78.1 ± 9.1 years and an average BMI value of 21.5 ± 4.3 kg/m^2^. All patients underwent dual-energy X-ray absorptiometry (DXA) of the contralateral hip; hip structural analysis (HSA) software was used to analyze the femoral geometry parameters, including hip axis length (HAL), neck-shaft angle (NSA), cross-sectional area (CSA), the cross-sectional moment of inertia (CSMI), the buckling ratio (BR), and cortical thickness.

**Results:**

The cortical thickness in the intertrochanteric region was reduced in the trochanteric fractures group compared to the femoral neck fracture group (*P* < 0.05). There were no statistically significant differences (*P* > 0.05) in gender, age, height, weight, or BMI between the two groups. In addition, no statistically significant differences (*P* > 0.05) were found in the CSA, CSMI, or BR of the femoral neck or the intertrochanteric region between the two groups. There were no statistically significant differences (*P* > 0.05) in femoral neck cortical thickness between the two groups.

**Conclusions:**

Cortical thickness thinning in the intertrochanteric region may be one of the relevant factors causing different types of hip fractures, especially in elderly patients.

## Background

With the escalation of socioeconomic development and an aging population, the number of middle aged and elderly people with hip fractures is becoming increasingly higher. As a common complication of osteoporosis, hip fractures are expected to affect up to 6.3 million individuals worldwide from 2050 and after, including 3.25 million in Asia [1]. Hip fractures are a leading cause of morbidity and mortality, with a high mortality rate of 30–33% one year after hip fracture occurrence [2]. Hence, studies assessing the relevant factors that cause hip fractures are urgently needed. Femoral neck and trochanteric fractures are two types of hip fractures. Previous studies have suggested that hip fractures can be easily caused by reduced hip bone mineral density (BMD) [3] and various features of the femoral geometry near the thigh bone in elderly people [4–9]. A bone density instrument was used for the measurements. For the lumbar spine measurement, the patient lay down on the center of the testing platform, the feet were put on the foot care, hip flexion was 90°, the lumbar intervertebral discs were perpendicular to the bed, and the spine was in the center of the bed and straight. Both sides of the iliac crests of T12 and L5 were displayed when scanning. For the hip measurement, the patient lay down on the center of the bed, the legs were straight and slightly outreached, inward rotation was 15–25°, and the femoral shaft was parallel to the long axis of the bed. However, most available studies have focused on comparing fracture and non-fracture groups, rarely assessing the differences between femoral neck and trochanteric fractures. In this study, the differences of femoral neck and trochanteric fractures were analyzed using dual-energy X-ray absorptiometry (DXA) data.

## Methods

### Patients

A total of 117 patients over the age of 50 with hip fractures resulting from low-energy falls were consecutively admitted to The Second Affiliated Hospital of Fujian Medical University from May 2013 to May 2014 and enrolled in this study. Cases with hip fractures resulting from severe trauma, such as car accidents and severe injuries, were excluded. Patients with secondary osteoporosis, including pathological fractures, glucocorticoid-induced osteoporosis (GIOP), and hyperparathyroidism, were excluded as well. These patients had not received osteoporosis treatments they were offered treatment and didn’t do it, because of their living standards and health awareness. Hip fractures can be classified into femoral neck and femoral trochanteric types. The femoral neck and trochanteric regions are within and outside of the articular capsule, respectively.

The 117 patients were divided into femoral neck and trochanteric fracture groups. There were 69 patients with femoral neck fractures, 20 men and 49 women with an average age of 75.1 ± 9.6 (50–97 years) and an average BMI value of 21.6 ± 4.1 kg/m^2^. The trochanteric group had 48 patients, 16 men and 32 women with an average age of 78.1 ± 9.1 (50–91 years) and an average BMI value of 21.5 ± 4.3 kg/m^2^.

### Measurement methods

For the BMD measurements, the DXA of the hip was performed on a Discovery A system (Hologic, Bedford, MA), with a coefficient of variation of 0.248% (after the bone density instrument was powered on every day, calibration was conducted, and daily quality control was performed using a dedicated vertebral body phantom; the measurement data were plotted according to the Shewhart control chart). BMD in the femoral neck region, femoral intertrochanteric region, femoral Ward’s region, femoral intertrochanteric region, and total hip region was measured on the uninjured side. Hip structural analysis (HSA) was used to assess the cortical thickness, cross-sectional area (CSA), cross-sectional moment of inertia (CSMI), buckling ratio (BR), hip axis length (HAL), and neck-shaft angle (NSA).

### Statistical Methods

The measurement data were expressed as mean ± standard deviation (SD). Comparison of the two groups was performed by an unpaired t-test. A homogeneity of variance test was done and a Chi-square test was used to evaluate the statistical data. The statistical analyses were performed with SPSS 17.0 software. *P* < 0.05 was considered statistically significant.

## Results

### General data

No statistically significant difference was found in the female to male ratios between the two groups (*p* = 0.652). In addition, there were no significant differences in both groups for age, height, weight, and BMI (*p* > 0.05; Table 1).

**Table 1:** Basic data in the two groups

	Femoral neck fracture	Trochanteric fracture	P(0.05)	t or χ2
Male/Female	20/49	16/32	0.652	0.202
Age(yrs)	75.1±9.60	78.1±9.10	0.114	1.592
Weight(Kg)	55.3±14.0	54.5±11.5	0.737	0.336
Height(m)	1.59±0.08	1.58±0.08	0.960	0.611
BMI	21.6±4.10	21.4±4.30	0.884	0.146

### BMD

There were no significant differences in BMD in the femoral neck region, femoral intertrochanteric region, femoral Ward’s region, femoral intertrochanteric region, and total hip region (*p* > 0.05; Table 2).

**Table 2:** Hip BMD values in the two groups

Site	Femoral neck fracture (g/cm2)	Trochanteric fracture (g/cm2)	P(0.05)	t
Neck	0.514±0.102	0.489±0.117	0.227	1.215
Troch	0.506±0.098	0.469±0.085	0.094	2.110
Ward’s	0.338±0.091	0.323±0.123	0.451	0.756
Inter	0.756±0.187	0.721±0.174	0.306	1.028
Total hip	0.648±0.135	0.615±0.133	0.200	1.288

### Geometrical and Structural parameters

There were no statistically significant differences in HAL and NSA between the two groups (*P* = 0.874 and *P* = 0.177, respectively). In addition, there were no significant differences in CSA, CSMI, and BR in the neck region (*p* > 0.05). There were also no significant differences in CSA, CSMI, and BR in the intertrochanteric region (*p* > 0.05). What’s more, there was no significant difference in neck cortical thickness between the two groups (*P* = 0.251). Interestingly, compared with the femoral neck fracture group, patients with trochanteric fractures had reduced cortical thickness in the intertrochanteric region (*P* = 0.013). Detail-level data are summarized in Table 3.

**Table 3:** Structural parameters of the proximal femur in both groups

Item	Femoral neck fracture	Trochanteric fracture	P(0.05)	t
HAL(mm)	106.2±8.4	106.4±7.2	0.874	0.158
NSA(°)	129.3±5.0	130.6±4.9	0.177	1.360
Neck				
NN-thick(mm)	1.19±0.23	1.14±0.25	0.251	1.155
NN-CSA(cm2)	2.11±0.44	2.02±0.53c	0.340	0.957
NN-CSMI(cm4)	2.12±0.75	2.08±1.02	0.771	0.292
NN-BR	17.57±4.21	18.16±4.69	0.481	0.707
Inter				
IT-thick(mm)	2.85±0.77	2.48±0.76	0.013	2.522
IT-CSA(cm2)	3.62±1.00	3.50±1.14	0.560	0.585
IT-CSMI(cm4)	11.28±4.88	11.01±5.74	0.778	0.283
IT-BR	12.47±3.33	13.53±4.19	0.132	1.519

## Discussion

Nowadays, the number of middle aged and elderly people with hip fractures is growing increasingly higher [10]. In one study, hip fractures reduced life expectancy by 25% compared to an age- and sex-matched general population, and the development of deficits in ADLs after a hip fracture resulted in substantial morbidity, mortality, and costs, making it critical to conduct more research on hip fractures [11]. Kanis et al. [12] reported that the reduction of each standard deviation in BMD increased the risk of fracture1.4 to 2.6 times. Some studies [7, 13, 14] have suggested that hip fracture risk is sharply increased by low BMD. In addition, differences in femoral geometry and the expansion of femoral geometry parameters, such as the neck-shaft angle [4, 7, 13], hip axis [15, 16], and the length of the femoral neck [17], are important risk factors. Previous studies have evaluated the performance of quantitative computed tomography (QCT) and a dedicated 3D image analysis tool [Medical Image Analysis Framework-Femur option (MIAF-Femur)] in differentiating hip fracture and non-hip fracture subjects [18].

What is the difference between patients with femoral neck fractures and those with trochanteric fractures? According to one study, compared with patients with femoral neck fractures, the trochanteric fracture group was older, performed fewer daily activities independently, and often used walking aids at home [19]. Mautalen et al. [20] summarized studies evaluating hip fractures, and found that patients with trochanteric fractures were older and had lower weight, height, and BMI than patients with femoral neck fractures. In this study, we found that the patients in the trochanteric fracture group were older; however, there were no statistically significant differences in height, weight, or BMI between the two groups. Other studies have found that with increasing age, BMD was reduced accordingly; compared with non-fracture patients, the decreased BMD weakened the hip bone, easily leading to femoral neck or trochanteric fractures [7, 13, 14]. Kim et al. [21] reported that patients with trochanteric fractures had lower BMD in the femoral neck region and femoral intertrochanteric region compared with the femoral fracture group. However, according to the data from the current study (see Table 2), there were no statistically significant differences of BMD in the femoral neck region, femoral intertrochanteric region, femoral Ward’s region, femoral intertrochanteric region, or total hip region between the two groups, which was corroborated by Maeda et al. [22] and Johannesdottir et al. [23]. Our previous study showed that BMD reduction was rather fast before patients reach 65, with the declining trend stagnating thereafter [5]. And our previous study showed that most patients with hip fractures are older than 65, indicating that BMD reduction after the age of 65 is not the major factor causing hip fractures. Regarding geometrical parameters, Pulkkinen et al. [24] demonstrated that patients with femoral neck fractures had larger NSAs, suggesting the latter to be a predictor for distinguishing hip fracture types, while neck axis length (NAL) between the two groups showed no marked difference. In contrast, Maeda et al. [22] and Panula et al. [25] found no significant differences in NSA, HAL, and NAL between the femoral neck and trochanteric fracture groups. This research found that there were no statistically significant differences in HAL and NSA between both groups. With respect to structural parameters, studies have suggested statistically significant differences between fracture and non-fracture groups, with smaller CSA and CSMI and larger BR in the fracture group than in the non-fracture group [7, 8, 26]. However, studies comparing different hip fracture types are rare [27, 28]. The current research found no statistically significant differences in the CSA, CSMI, and BR of the femoral neck or intertrochanteric regions.

The cortical bone contributes to bone strength; a total cancellous bone removal from the femoral neck results in its strength declining at a rate of less than 10%, which indicates the cortical bone’s major role in strengthening the femoral neck [29]. With increasing age, the cortical bone becomes thinner [5, 30]. Ward et al. [31] found that in adults above 50, the cortical bone decreased in thickness at a rate of 14% every 10 years. Multiple studies [4–9, 24, 26] have suggested that a cortical thickness decrease can result in fragility fractures. Zebaze et al. [32] reported that the cancellous bone reduction rate was higher than that of the cortical bone during female menopause at the age of 50–64. In patients aged 65–79, cortical bone loss was highest, while after 80, 90% of the bone loss was from the cortical bone. In this study, 106 patients older than 66 had hip fractures, accounting for 90.6% of all the patients; this finding demonstrates that the patients with hip fractures were in the age group where the cortical bone became thinner. Maeda et al. [22] and Kim et al. [21] found that the cortical bone index in the femoral isthmus of trochanteric fractures was lower than that of femoral neck fractures. As shown in Table 3, a statistically significant difference was found in the cortical thickness between femoral neck and trochanteric fractures, with lower values obtained for cortical thickness in the femoral intertrochanteric region of trochanteric fractures. This indicates that trochanteric fractures are related to low cortical thickness, causing bone strength degradation. When there was pressure against the hip, the stress fell on the medial-inferior femoral neck, while the outer-upper femoral neck quadrant constituted the tension zone. Multiple studies [23, 33, 34] have revealed that the posterior-lower part of the femoral neck is the main area where cortical thickness decreases, with femoral neck fractures occurring. However, the current study found no significant difference in the cortical thickness of the femoral neck between the two groups. Indeed, the change in the cortical bone was not only reflected in its thickness but also affected its inner structure. It is possible that structural changes of the cortical bone differed between the two hip fracture types. Other than geometrical and structural factors, there must be other parameters causing hip fractures, which require further assessment by applying other methods.

We did not plan to do CT scans in the beginning, so we cannot provide CT scans of the hip and cannot support this study’s data with CT scans. We will conduct CT scans in future studies.

## Conclusions

This study indicates that cortical thickness reduction is one of the relevant factors leading to different hip fracture types, especially in elderly patients.

## Abbreviations

BMD: Bone mineral density
BMI: Body mass index
BR: Buckling ratio
CSA: Cross-sectional area
CSMI: Cross-sectional moment of inertia
DEXA: Dual energy x ray absorptiometry
DXA: Dual-energy X-ray absorptiometry
HAL: Hip axis length
HSA: Hip Structural Analysis
NSA: Neck-shaft angle
SD: Standard deviation
